# Preoperative short-course radiotherapy and long-course radiochemotherapy for locally advanced rectal cancer: Meta-analysis with trial sequential analysis of long-term survival data

**DOI:** 10.1371/journal.pone.0200142

**Published:** 2018-07-12

**Authors:** Xin Wang, Bobo Zheng, Xinlan Lu, Ruhai Bai, Linlin Feng, Quan Wang, Yan Zhao, Shuixiang He

**Affiliations:** 1 Department of Gastroenterology, First Affiliated Hospital of Xi’an Jiaotong University, Xi’an, Shaanxi, China; 2 Department of Gastrointestinal Surgery, West China Hospital, Sichuan University, Chengdu, Sichuan, China; 3 Global Health Institute, Xi’an Jiaotong University Health Science Center, Xi’an, Shaanxi, China; 4 Medical Imaging Center, Northwest Women’s and Children’s Hospital, Xi’an, Shaanxi, China; 5 Digestive Disease Hospital, Xijing Hospital, The Fourth Military Medical University, Xi'an, Shaanxi, China; University of Nebraska Medical Center, UNITED STATES

## Abstract

**Background and purpose:**

The role of preoperative short-course radiotherapy (SCRT) in rectal cancer treatment, when compared to long-course radiochemotherapy (LCRT), is still controversial. Thus the meta-analysis with trial sequential analysis (TSA) was performed to evaluate the long-term survival of SCRT and LCRT as therapeutic regimens for locally advanced rectal cancer.

**Material and methods:**

PubMed, Embase, and the Cochrane Central Register of Controlled Trials were searched up to August 2017 for eligible studies. Hazard ratios (HRs) or odds ratios (ORs) of overall survival (OS), disease free survival (DFS) and local recurrence (LR) with the corresponding 95% confidence intervals (CIs) were calculated and TSA was applied.

**Results:**

11 studies with 1984 patients were included. There was no significant difference in OS (HR = 0.92, 95% CI: 0.75–1.13, *p* = 0.44), DFS (HR = 0.94, 95% CI: 0.79–1.12, *p* = 0.50) and LR (OR = 0.73, 95% CI: 0.49–1.08, *p* = 0.11) between SCRT and LCRT groups. TSA suggested firm evidence for lacking on average a -10% relative risk reduction (RRR) in 4-year OS but no statistical significance in 4-year DFS.

**Conclusions:**

Preoperative SCRT is as effective as LCRT for locally advanced colorectal cancer in long-term survival. SCRT could be preferential while facing long waiting lists or lacking medical resource.

## Introduction

Preoperative radiotherapy has been shown conclusively to improve local control for rectal cancer [1–2]. For locally advanced stage II-III resectable rectal cancer (mostly cT3 without threatened or involved mesorectal fascia), either preoperative short-course radiotherapy (SCRT) of 25 Gy in 5 consecutive days or long-course chemoradiotherapy (LCRT) (45-50Gy, 1.8-2Gy/fr with concomitant 5-FU-based chemotherapy) followed by radical Total Mesorectal Excision is recommended [3–4]. The benefit of SCRT, as proposed by Swedish Rectal Cancer Trial [5], is a lower rate of early toxicity when compared to chemoradiation [6–8]. Short-course irradiation reduced the risk of local recurrence (LR) by half and showed evident overall survival (OS) improvement [9]. Short-course schedule is less expensive and more convenient as well, especially in centers with long waiting lists [10]. The superiority of LCRT, as proposed by Sauer [6], was demonstrated in comparison to postoperative chemoradiotherapy in terms of local control.

Although both SCRT and LCRT have been practiced in parallel for more than 20 years, it is not clear which form of preoperative radiotherapy provides better tumor control and long term outcomes. Two meta-analyses [11–12] showed that, in terms of sphincter preservation rate, LR rate, grade 3–4 acute toxicity, R0 resection rate and downstaging rate, SCRT is as effective as LCRT for management of rectal cancer. A randomized controlled trial (RCT) [13] reported that The 5-year disease free survival (DFS) and OS were significantly better in the LCRT group than the SCRT group. However, some other RCTs [10,14–15] showed that there was no significant difference in local control and OS between SCRT and LCRT groups.

Based on this situation, we performed meta-analyses to evaluate the long-term prognoses of preoperative SCRT and LCRT as the therapeutic regimens for locally advanced rectal cancer. However, meta-analysis may obtain false positive results (type I errors) or overestimate treatment effects due to systematic errors (bias) and random errors (play of chance). So we performed Trial sequential analysis (TSA) as well, which combines a priori information size calculation for a meta-analysis with the adaptation of monitoring boundaries to evaluate the accumulating data [16–17]. Therefore, we carried out this meta-analysis with TSA to investigate long-term outcomes of the SCRT and LCRT regimens for rectal cancer.

## Methods

### Literature search

A comprehensive literature search in Pubmed, Embase, and the Cochrane Library databases up to August 2017 was conducted to identify relevant literatures. The search was based on different combinations of the following terms: “rectal cancer”, “long-course chemoradiotherapy”, “preoperative chemoradiotherapy”, “conventional chemoradiotherapy”, “radiotherapy”, “survival” and “short-course radiotherapy”. In addition, references cited in the relevant review articles and meta-analyses were also checked for potentially eligible studies. There was no other limit imposed on this search. PRISMA statement and guidelines [18] were consulted during the stages of design, analysis, and reporting of this meta-analysis (PRISMA Checklist is available in S1 File).

### Inclusion and exclusion criteria

(1) Studies that compared SCRT with LCRT in the treatment of locally advanced rectal cancer with follow-up of at least 2 years. (2) Studies that evaluated at least one of the three primary outcomes (OS, DFS or LR). (3) In cases of duplicates, the most recent study was included. (4) No language limitation was imposed. (5) Case reports, review articles and letters were excluded.

### Selection and quality assessment

Two reviewers independently screened the titles, abstracts and full texts to determine whether the studies met the inclusion criteria, then assessed the qualities of the eligible studies and extracted data, discrepancies were resolved by consensus. The modified Jadad scale, developed by Greenhalgh [19] and Oremus [20], was used to assess the methodological quality of the included studies [21]. Specifically, the modified version of the Jadad scale consists of three additional questions for the 6-item Jadad scale: (1) was there a clear description of the inclusion/exclusion criteria? (2) was the method used to assess adverse effects described? (3) were the methods of statistical analysis described? One point would be awarded for each affirmative response, while no point would be awarded for a negative response. Scale scores ranged from 0 to 8 points, with higher scores indicating better quality [21]. In addition, to evaluate the pooled results, the Grading of Recommendations Assessment, Development, and Evaluation system (GRADE system) was employed to rate the quality of the evidence for each outcome [22].

### Data extraction and synthesis

The extracted contents included: General study information (such as title, publication year, and first author), characteristics of participants and diseases, interventions (such as patients’ age and sex, type of study, sample size, interventions, tumor stage, length of follow-up) and outcomes (OS, DFS and LR).

### Statistical analysis

For the time-to-event endpoints (OS and DFS), hazard ratios (HRs) with their corresponding 95% confidence intervals (CIs) were combined as the effective value to assess the summary effects. The HRs and their 95% CIs were extracted explicitly from the included articles or calculated from the available numerical data using methods reported by Parmar [23], and calculated following the method developed by Tierney [24]. In addition, for LR, odds ratios (OR) with 95% CIs were calculated using the Mantel-Haenszel method.

Meta-analyses were performed using Review Manager Software version 5.2 (Cochrane Collaboration). The fixed-effect and random-effect models were used to calculate the outcomes, and the *p*-values less than 0.05 were considered statistically significant. In case of significant statistical heterogeneity, only the results from the random effect model were reported. Heterogeneity among the trials was determined by means of the Cochran *Q* value and quantified using the *I*^*2*^ inconsistency test. Subgroup analyses were performed to compare outcomes from RCT and non RCT respectively.

### Trial sequential analysis

A required diversity (*D*^*2*^)-adjusted information size was calculated, with *D*^*2*^ being the relative variance reduction when the meta-analysis model was changed from a random-effect into a fixed-effect model [25]. *D*^*2*^ represents the percentage that the variability between trials, consists of the sum of the between-trial variability and a sampling error estimate considering the required information size. *D*^*2*^ differs from inconsistency (*I*^*2*^), which is the intuitively obvious adjusting factor based on the common quantification of heterogeneity, also which underestimates the required information size [25].

TSA was performed with a desire to maintain an overall 5% risk of type I error, being the standard in most meta-analyses and systematic reviews. In addition, the required information size was calculated (an alpha error of 5%, a beta error of 20%) [16,26–27]. Theoretically, If the trial sequential monitoring boundary is crossed before the required information size is reached, firm evidence may be established. However, if the boundary is not surpassed, it is most probably necessary to continue doing trials. Trial sequential analysis version 0.9 beta was used for all these analyses.

## Results

### Literature search

**Fig 1:**
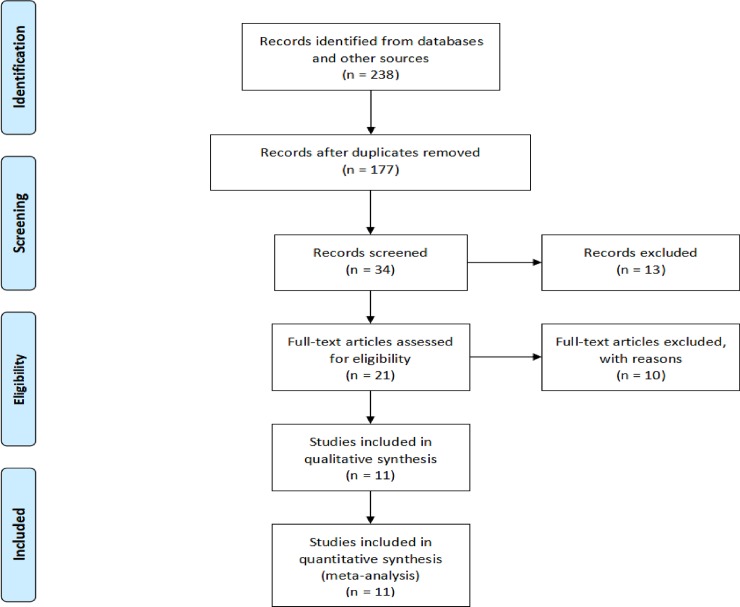
Flowchart of eligible studies identification. 11 studies (4 RCTs and 7 non-RCTs) with a total of 1984 patients were included in this meta-analysis.

As shown in Fig 1, initially about 238 articles were searched from the databases up to August 2017. Based on the inclusion and exclusion criteria, 217 studies were excluded and 21 studies were subjected to a more detailed review. Finally, 11 studies (4 RCTs [10,13–15] and 7 non-RCTs [28–34]) with a total of 1984 patients were included in this meta-analysis.

### Study characteristics and methodological quality

Table 1 and S1 Table listed the main characteristics of the 11 studies. The sample sizes in the studies ranged from 29 to 427. 11 included studies all analyzed OS and DFS, and 10 of them analyzed LR. 4 RCTs [10,13–15] out of the 11 studies earned scores of 6 for quality assessment based on the modified Jadad scale, and the 7 non-RCTs [28–34] were scored 4.

**Table 1:** Characteristics and Jadad scores of included studies.

Study	Country	Study type	No. of patients		Sex, F/M		Age		DBTA, cm No. of patients		Follow up, month	Jadad score
			SCRT	LCRT	SCRT	LCRT	SCRT	LCRT	SCRT	LCRT		
Bujko 2006[10]	Poland	RCT	155	157	55/100	54/103	60(30–75)^#^	59(34–73)^#^	5.8(2–10)^#^	5.7(2–9)^#^	48(31–69)^#^	6
Kairevičė L 2017[13]	Lithuania	RCT	68	72	25/43	22/50	66.5±9.5*	63.14±10.1*	U:5 M:29 L:34	U:5 M:37 L:30	60.5(5–108)^#^	6
Ngan SY 2012[14]	Australia and New Zealand	RCT	162	161	45/117	41/120	63(26–80)^#^	64(29–82)^#^	U:26 M:88 L:48	U:42 M:88 L:31	70.8(36–93.6)^#^	6
Eitta MA 2010[15]	Egypt	RCT	14	15	5/9	5/10	53(32–75)^#^	45(25–65)^#^	U:0 M:3 L:11	U:0 M:2 L:13	18(6–28)^#^	6
Guckenberger M 2012[29]	Würzburg	PS	108	107	32/76	27/80	64*	66*	U:9 M:53 L:46	U:4 M:25 L:78	49(3–138)^#^	4
Beppu N 2015[28]	Japan	RS	104	61	32/72	16/45	61(39–85)^#^	63(34–79)^#^	U:0 M:49 L:55	U:0 M:28 L:33	44(12–85)^#^	4
Krajcovicova I 2012[30]	Slovak Republic	RS	96	55	33/63	15/40	F:63(36–84)^#^ M:61(29–83)^#^	F:58(42–72)^#^ M:62(49–78)^#^	NR	NR	48(2–128)^#^	4
Yeh CH 2012[31]	Taiwan	RS	28	37	11/17	13/24	67(42–87)^#^	60(30–87)^#^	U:10 M:7 L:11	U:7 M:9 L:21	36(3.12–61.92)^#^	4
Inoue Y 2011[32]	Japan	RS	51	22	NR	NR	NR	NR	NR	NR	49*	4
Klenova A 2007[33]	Bulgaria	RS	51	33	21/30	13/20	NR	NR	U:0 M:19 L:32	U:0 M:12 L:21	53(22–84)^#^	4
Abdel-Rahman O 2017[34]	Egypt and Canada	RS	241	186	89/152	54/132	67*	62*	NR	NR	NR	4

* values are mean±standard deviation

^#^ values are median (range)

SCRT: short-course radiotherapy, LCRT: long-course radiochemotherapy

DBTA: Distance between tumour and anal verge

RCT: randomized controlled trials, RS: retrospective study, PS: prospective study

U: high rectal cancer, M: middle rectal cancer, L: low rectal cancer

NR: not reported.

### Overall survival

**Fig 2:**
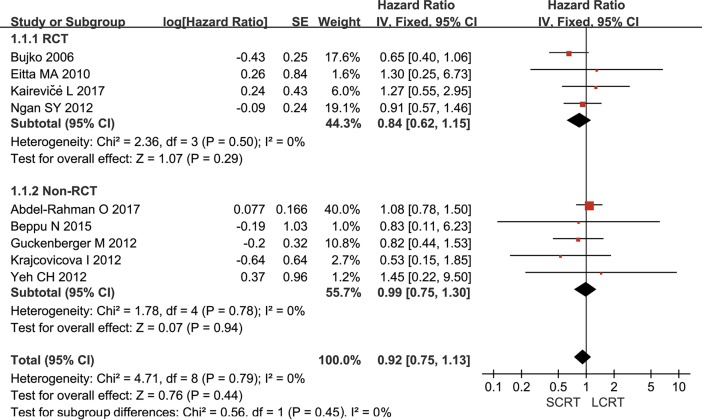
Meta-analysis of cumulative overall survival. There was no significant difference in OS between SCRT and LCRT groups (HR = 0.92, 95% CI: 0.75–1.13, *p* = 0.44). The subgroup analysis of RCTs or non-RCTs found similar results.

All included studies provided OS information, however, 2 [32–33] studies did not provide HR information. Klenova A [33] reported that the 4-year OS were 72% and 70% in SCRT and LCRT groups, respectively. Inoue Y [32] showed that the 5-year OS rate were 83.3% (LCRT) versus 83.4% (SCRT). Meta-analysis of the available data pooled by fixed-effect model showed that the result of the heterogeneity test was *p* = 0.79/*I*^*2*^ = 0%, and no significant difference was observed in OS between SCRT and LCRT groups (HR = 0.92, 95% CI: 0.75–1.13, *p* = 0.44) (Fig 2). The subgroup analysis of RCTs or non-RCTs showed similar results. The specific information of OS for the first 5 years was summarized in S2 Table.

### Disease free survival

**Fig 3:**
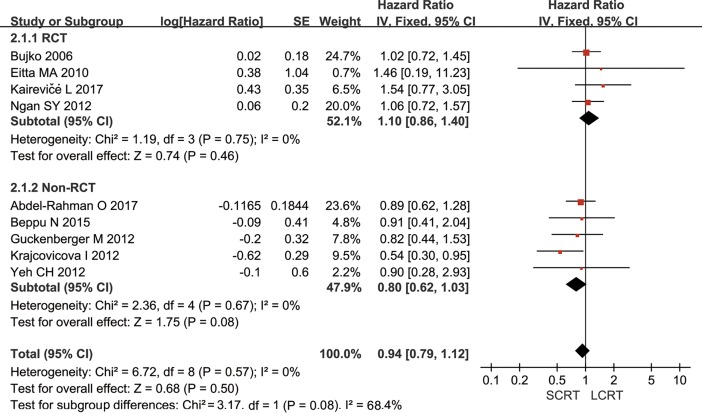
Meta-analysis of cumulative disease free survival. No significant difference was found (HR = 0.94, 95% CI: 0.79–1.12, *p* = 0.50) in disease free survival. Subgroup analysis showed that the difference remained insignificant when RCTs and non-RCTs were analyzed separately.

10 out of 11 studies reported DFS. Inoue Y [32] provided that the 4-year DFS were 66% and 68% in SCRT and LCRT groups, respectively. However, no information for obtaining HR was provided. Meta-analysis of 4 RCTs and 5 non-RCTs using the fixed-effect model demonstrated that the heterogeneity between studies was *p* = 0.57/*I*^*2*^ = 0% and no significant difference was found (HR = 0.94, 95% CI: 0.79–1.12, *p* = 0.50). Subgroup analysis showed that the difference remained insignificant when RCTs and non-RCTs were analyzed separately (Fig 3). The specific information of DFS for the first 5 years was summarized in S3 Table.

### Local recurrence and distant recurrence

**Fig 4:**
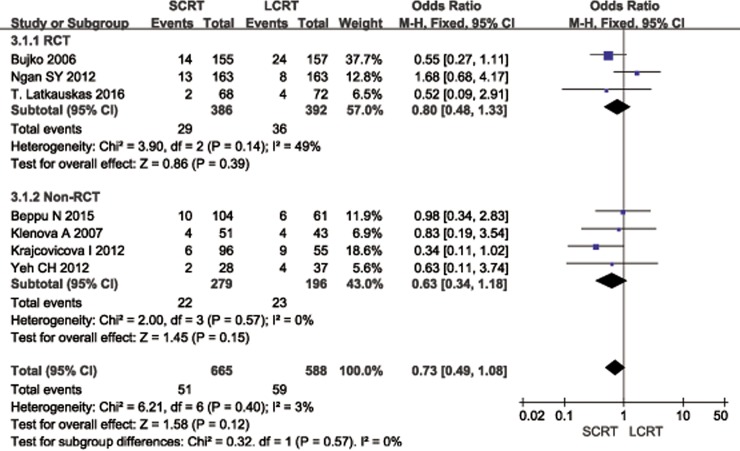
Meta-analysis of 3-year local recurrence. There was no difference between CRT and LCRT (OR = 0.73, 95% CI: 0.49–1.08, *p* = 0.11) in 3-year local recurrence. Subgroup analysis found no significant difference in either RCTs or non-RCTs as well.

The data of 3-year LR was reported in 3 RCTs [10,13–14] and 4 non-RCTs [28,30–31,33]. Meta-analysis of the data reported no difference between SCRT and LCRT in terms of LR (OR = 0.73, 95% CI: 0.49–1.08, *p* = 0.11). Subgroup analyses of either RCTs or non-RCTs both showed similar results (Fig 4). The specific information of LR for the first 5 years was summarized in S4 Table.

Bujko K [10] showed that the crude incidence of distant metastasis was 31.4% in the short-course group and 34.6% in the chemoradiation group (*p* = 0.54). Ngan SY [14] reported that 5-year distant recurrence rates were 27% for SCRT and 30% for LCRT (HR (LCRT:SCRT) = 1.04, 95% CI: 0.69–1.56, *p* = 0.92). A RCT [13] reported that distant metastases developed in 14 (21.9%) cases after SCRT and in 9 (12.7%) cases after LCRT (*p* > 0.05) during the follow-up of 39.7 months, and the HR of distant metastasis for SCRT patients compared to LCRT patients was 2.2 (95% CI: 0.95–5.10). Yeh CH [31] showed that the distant metastasis rates of patients received SCRT and LCRT were 31.5% and 31.1% (*p* = 0.21), respectively.

### Trial sequential analyses

**Fig 5:**
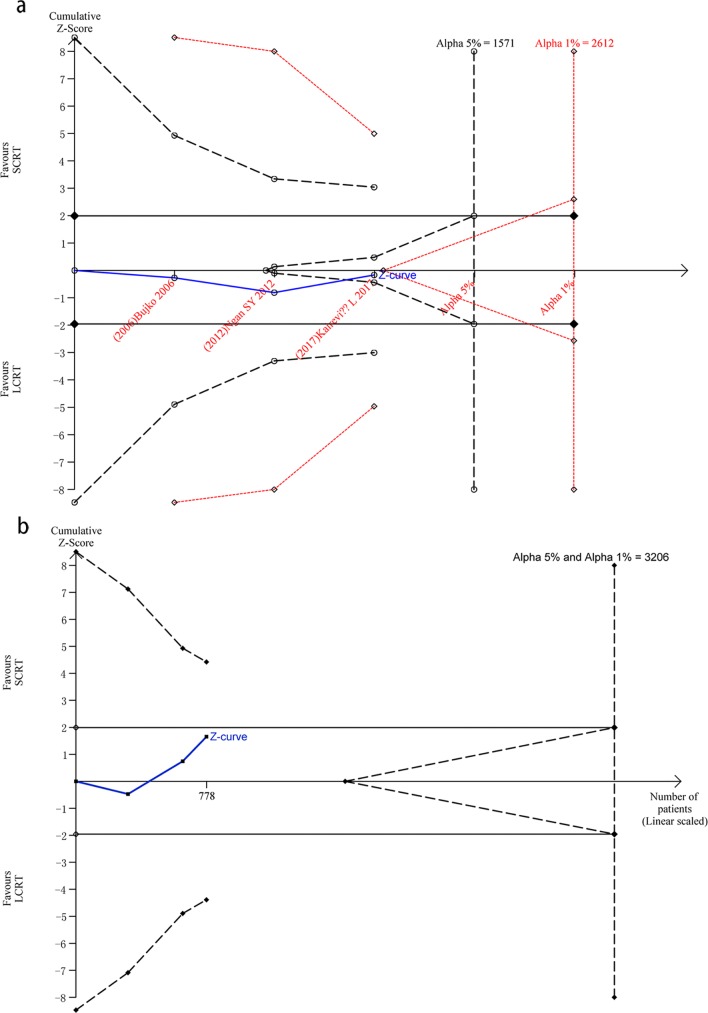
Trial sequential analysis of 4-year overall survival. 5a, Trial sequential analysis of 4-year overall survival. The required heterogeneity-adjusted information size using 5% risk of type I error and 20% risk of type II error. The cumulative z curve crossed the futility boundary, suggesting firm evidence for lack of on average a -10% relative risk reduction in 4-year OS. **5b,** Trial sequential analysis of 4-year disease free survival. When compared with LCRT treatment in 4-year DFS, neither the traditional boundary nor the trial sequential monitoring boundary was crossed for a -10% relative risk reduction with SCRT. In addition, the futility boundary was not crossed.

We conducted trial sequential analyses using the information size adjusting for the presence of heterogeneity based on three RCTs [10,13–14] with modified Jadad scale score of at least 6. The required heterogeneity-adjusted information size with 5% risk of type I error (risk of obtaining a false ‘positive’ result), 20% risk of type II error (risk of obtaining false ‘negative’ result) and an anticipated RR = 1.1 (i.e. a relative risk reduction in 4-year OS by SCRT of -10%) was calculated to 1571 patients. The cumulative z curve crossed the futility boundary, suggesting firm evidence for lack of on average a -10% RRR in 4-year OS. The required information size using 1% risk of type I error instead was 2612 patients. The analyses did not yield any sign of statistical significance whatsoever. The cumulative z curve crossed neither the traditional boundary nor the trial sequential monitoring boundary but was very close to the futility boundaries, suggesting a lack of firm evidence for a -10% RRR in the SCRT group compared to the LCRT group regarding to 4-year OS (Fig 5A).

In addition, we performed trial sequential analysis for 4-year DFS with a type I error of 5% and 1%, type II error of 20% (80% power), and adjusted for heterogeneity among included trials [10,13–14]. When compared with LCRT treatment, neither the traditional boundary nor the trial sequential monitoring boundary was crossed for a -10% RRR with SCRT. In addition, the futility boundary was not crossed (Fig 5B), suggesting the need for more evidence to establish additional benefits of SCRT over LCRT treatment.

### Evidence rating of the critical outcomes

The GRADE system was used to synthesize and rate the evidence for each outcome, and the quality of evidence was summarized in Table 2. The overall qualities of evidence for those outcomes were of high quality. Hence, further research is unlikely to change our confidence in the estimate of effect.

**Table 2:** Quality of evidence for each outcome using GRADE system.

Outcome	Study design	Studies (participants)	Quality assessment						Summary of findings		
			Risk of bias	Inconsistency	Indirectness	Imprecision	Publication bias	Overall quality of evidence	HR or OR (95%CI)	Heterogeneity	
										I^2^ (%)	p value
OS		9(1895)							0.92 (0.75–1.13)	0	0.79
	RCT	3(778)	No serious	No serious	No serious	No serious	Undetected	⊕⊕⊕⊕ high	0.84 (0.62–1.15)	0	0.50
	Non-RCT	6(1117)	No serious	No serious	No serious	No serious	Undetected	⊕⊕⊝⊝low	0.99 (0.75–1.30)	0	0.78
DFS		9(1895)							0.94 (0.79–1.12)	0	0.57
	RCT	3(778)	No serious	No serious	No serious	No serious	Undetected	⊕⊕⊕⊕ high	1.10 (0.86–1.40)	0	0.75
	Non-RCT	6(1117)	No serious	No serious	No serious	No serious	Undetected	⊕⊕⊝⊝low	0.80 (0.62–1.03)	0	0.87
LR		7(1250)							0.73 (0.49–1.08)	3	0.41
	RCT	3(775)	No serious	No serious	No serious	No serious	Undetected	⊕⊕⊕⊕ high	0.80 (0.48–1.33)	48	0.15
	Non-RCT	4(475)	No serious	No serious	No serious	No serious	Undetected	⊕⊕⊝⊝low	0.63 (0.34–1.18)	0	0.57

## Discussion

According to the updated ESMO clinical practice guidelines of 2017, both LCRT and SCRT for resectable locally advanced rectal cancer are recommended [3]. However, due to the various routine deliveries of neoadjuvant treatment regimens, different treatment strategies were adopted among countries or even in the same country.

Several meta-analyses [11–12,35–37] compared these two different preoperative treatment regimens and found no difference in DFS and OS. Among them, 3 meta-analyses reported that LCRT resulted in significantly lower LR rate, but the recent 2 meta-analyses [11–12] showed that no significant difference in LR rate between the two regimens. All systematic reviews and meta-analyses made a consensus that LCRT resulted in significantly higher pathological complete response rate and higher acute toxicity.

The present meta-analysis investigated long-term outcomes of the SCRT versus LCRT for advanced rectal cancer. OS, DFS and LR were not significantly different between the patients treated using the SCRT and the LCRT regimens. However, the prevalence of meta-analysis at risk of random error due to repetitive testing seemed too high to be ignored. TSA might eliminate early false positive findings due to imprecision and repeated significance testing in meta-analyses. And TSA could also provide a required diversity adjusted information size, a threshold for a statistically significant treatment effect, and the threshold for futility [26,38]. We therefore undertook a trial sequential analysis to consolidate the available literature.

Presented by a systematic overview of radiotherapy in rectal cancer, statistically lower LR rates were observed in most trials comparing preoperative radiotherapy (followed by surgery) versus surgery alone [39]. In the Australia and New Zealand RCT [14], there was no statistically significant difference in LR rate between preoperative SCRT and LCRT radiotherapy, although the trend favored LC (3-year LR rates: 10% in SC group and 2% in LC group; inferred 95% CI for SCRT-LCRT approximately -2.1% to 8.3%). However, our present study demonstrated a small difference in 3-year LR rate (8.8%), favoring SCRT. The 95% CI (SCRT:LCRT) was 0.49 to 1.08, including differences of 3.2% or more, in favor of SCRT (i.e. 6.8% vs 10%). But the trial did not exclude the potential important clinical difference in 3-year LR rates.

Kapiteijn E [39] considered that LR was significantly related to the distance from the anal verge. This may be due to that 82.8% of patients had a tumor in the lower third. However, no significant difference was found in terms of LR in the present meta-analysis. The data was consistent with either no difference or an important clinical difference in favor of SCRT, and it’s unlikely that there was an important difference favoring LCRT.

Latkauskas T [40] considered that the death of rectal cancer was mainly correlated with distant metastases, but not LR. This could be the explaination for the trivial survival benefit reported by most trials, while comparing short-term with long course neoadjuvant treatment for rectal cancer. However, 3 RCTs [10,13–14] reported that no significant difference was found in incidence of distant metastases between SCRT and LCRT groups. The France FFCD 9203 did not certify any superiority for the addition of 5-Fu to RT in terms of either DFS or OS, when comparing preoperative radiotherapy with chemoradiotherapy [41]. According to the results of the Lithuania RCT [40], 3-year DFS was better in LCRT group compared to SCRT group without significant difference in OS. Surgical recovery and perioperative morbidity were similar between the groups. Kairevičė L [13] reported that the 5-year DFS and OS were significantly better in the LCRT group than that in the SCRT group. However, the study was based on a small number of patients, and this could be one of its biggest limitations. Data on positive pathological lymph nodes in Lithuania RCT [40] -25 (36.8%) cases in the SCRT group and 18 (25%) cases in CRT group (*p* > 0.05)—could tell that there was an imbalance in the original nodal status between both arms.

While the current study is focused on comparing LCRT and SCRT, the use of sequential chemotherapy in SCRT has a role in the long-term survival. A population-based cohort study recruited 123 patients who were with stage II rectal cancer and received preoperative SCRT plus chemotherapy, and its subgroup analysis suggested that adjuvant chemotherapy improved DFS (HR:0.24; 95% CI:0.07–0.85; *p* = 0.027) and OS (HR = 0.22; 95% CI: 0.069–0.70; *p* = 0.011) in patients with ≥ 2 risk features [42]. A phase II trial, which included 50 patients with stage VI rectal cancer, demonstrated a clear OS advantage with SCRT plus chemotherapy (*p* = 0.004) [43]. Multicenter RCTs are needed to confirm this advantage.

For the TSA of 4-year OS, the calculated diversity-adjusted required information size (DARIS) was 1571 participants, considerating the patient proportion in the control group with the outcome of 5.28%, a RRR of 20%, an alpha of 5%, a beta of 20% (the required information size using 1% risk of type I error is 2612 patients instead). 35.9% of the DARIS has been reached after accruing the patients to altogether 564 from 3 RCTs. The cumulative z curve crossed the futility boundary. We also performed TSA for 4-year DFS with a type I error of 5% and 1%, type II error of 20% (80% power). The required information size using 1% or 5% risk of type I error was 3206 patients. Accordingly, with 483 accrued participants in 3 RCTs, only 15.1% of the DARIS had been reached.

Considering medical expenses, Beppu N [28] reported that the SCRT regimen cost about $2,650 while the LCRT regimen cost about $7,050. Therefore, convenience and a lower cost were the major benefits of short term treatment. In addition, when considering patient convenience, the waiting period was about half a month for patients who received SCRT, while it was > 3 months for LCRT regimen recipients. SCRT could be the preferential treatment for locally advanced colorectal cancer while facing long waiting lists or lack of medical resource.

### Limitation

First of all, though all included studies earned more than scores of 4 for quality, all RCT exist some potential risks of bias. Only 3 out of all included RCTS reported the method of adequate randomized sequence generation [10,13–14], the other RCT showed unclear randomization [15]. This could have an influence on diagnostic procedures, RT and surgical techniques, as well as survival results. Moreover, the trial sequential analyses demonstrated that we have never had convincing evidence in favor of SCRT over LCRT for rectal cancer and we are still far from having it.

In conclusion, the present study demonstrated that preoperative SCRT is as effective as LCRT for the treatment of rectal cancer in terms of OS, DFS and LR. Further adequately powered trials with lower risk of bias are necessary to provide more robust evidence.

## Supporting information

S1 File PRISMA 2009 checklist. (PDF)

S1 Table Summary of interventions used in included studies. (DOC)

S2 Table Summary of overall survival information in included studies. (DOC)

S3 Table Summary of disease free survival information in included studies. (DOC)

S4 Table Summary of local recurrence information in included studies. (DOC)
