# Supplementary material for: Preoperative short-course radiotherapy and long-course radiochemotherapy for locally advanced rectal cancer: Meta-analysis with trial sequential analysis of long-term survival data
Source: PLoS One. 2018 Jul 12;13(7):e0200142. doi: 10.1371/journal.pone.0200142 (PMC6042715; doi:10.1371/journal.pone.0200142)
Supplement: S1 Table — (DOC) [file pone.0200142.s002.doc]

**S1 Table. Summary of interventions used in included studies**

| **Study** | **Cancer stage** | **Preoperative interventions** | | **Surgery** | **Surgery time**  **after Preoperative interventions** | | **Postoperative chemotherapy** | |
| --- | --- | --- | --- | --- | --- | --- | --- | --- |
|  |  | **SCRT** | **LCRT** |  | **SCRT** | **LCRT** | **SCRT** | **LCRT** |
| Bujko 2006[10] | cT3-4  N0-2 | 25 Gy in 5 fractions RT, in 1 week | 50.4 Gy in 28 fractions RT, with 5-FU and lv on the 1st and the 5th week | TME | in 1 week | 4-6 weeks | 6 months of 5-FU and Lv | 4 months of 5-FU and Lv |
| Klenova A 2007[33] | T2-4 | 25 Gy in 5 fractions (5 Gy daily) RT, for 5 days | 50 Gy in 25 fractions (2 Gy daily) RT, in 5 weeks | TME | 3-5 days | 4-5 weeks | 4-6 cycles of 5-FU and Lv for 5 days, every 4 weeks, for patients with pT4 and/or pN+ | |
| Eitta MA 2010[15] | cT2-4  N0-2 | 25 Gy in 5 fractions RT, over 1 week | 45Gy in 25 fractions RT, over 5 weeks | APR  or LAR | in 1 week | 4-6 weeks | Mayo Clinic (low risk patients) or FOLFOX bolus (high risk patients), 4-6 weeks after surgery. | |
| Inoue Y 2011[32] | T2-4 | 20 Gy in 4 fractions RT, with  5FU based chemotherapy | 45-50Gy long course RT, with 5FU based chemotherapy | NR | immediate surgery | delayed surgery | NR | NR |
| Ngan SY 2012[14] | cT3 | 25 Gy in 5 fractions RT, in 1 week | 50.4 Gy in 28 fractions RT, over 5weeks and 3 days, with continuous 5-FU | APR or  non APR | 3-7 days | 4-6 weeks | 6 monthly courses of 5-FU and folinic acid for 5 days, commenced 4-6 weeks after surgery | 4 monthly courses of 5-FU and folinic acid for 5 days, commenced 4-6 weeks post surgery |
| Guckenberger M 2012[29] | T2-4 | 29 Gy in 10 fractions RT, twice daily for 5 days. Adjuvant 5-FU-based chemotherapy for pathological stage UICC≥II patients. | 50.4 Gy in 28 fractions RT, over 5.5 weeks, with continuous 5-FU or 5-FU and ox­aliplatin. | TME | in 1 week | 4-6 weeks | NR | NR |
| Krajcovicova I 2012[30] | T2-4  N1-2 | 25 Gy (5 × 5.0 Gy per week) RT | 45–46 Gy (1.8-2.0 Gy per day) RT, with 5-FU and Lv on the 1st and the 5th week, or capecitabine | APR or phincter-  preservation. | 5.9(1-60) days^#^ | 42(17-72) days^#^ | NR | NR |
| Yeh CH 2012[31] | T2-4  N1-2 | 25 Gy in 5 fractions RT, 5 times per week. | 50.4 Gy in 28 fractions RT, 5 times per week. with 5-FU and Lv on the 1st and the 5th week. | TME | in 1 week | 4-6 weeks | 4 months of 5-FU and Lv for patients with stage II–III  disease after surgery | |
| Beppu N 2015[28] | T0-4  N1-2 | 25 Gy in 10 fractions RT, twice daily for 5 days. | 45 Gy in 25 fractions RT over 5 weeks, with Irinotecan on days 1, 8, 22, and 29. | TME | 4 weeks | 6-10 weeks | NR | NR |
| Kairevičė L 2017[13] | cT3-4  N0-2 | 25 Gy in 5 fractions RT, in 5 days | 50Gy in 25 fractions RT, over 5weeks, with 5-FU and lv on the 1st and the 5th week | TME | 6-8 weeks | 6-8 weeks | 4 cycles of 5-FU and Lv for 5 days, every 4 weeks, within 8 weeks after surgery | |
| Abdel-Rahman O 2017[34] | T0-4  N0-2 | 25 Gy in 5 fractions RT | 45-50.4 Gy in conventional fractionations RT | APR or LAR | 1.5 (0.14-27.14) weeks^#^ | 5.645 (0.14-106.43) weeks^#^ | Adjuvant chemotherapy | |

^#^ values are median(range)

SCRT: short-course radiotherapy, LCRT: long-course radiochemotherapy, RT: radiotherapy;

5-FU: fluorouracil, Lv: leucovorin;

TME: total mesorectal excision; APR: abdomino-perineal resection, LAR :low anterior resection.

H: High-quality;

NR: not reported.
