# Supplementary material for: Preoperative short-course radiotherapy and long-course radiochemotherapy for locally advanced rectal cancer: Meta-analysis with trial sequential analysis of long-term survival data
Source: PLoS One. 2018 Jul 12;13(7):e0200142. doi: 10.1371/journal.pone.0200142 (PMC6042715; doi:10.1371/journal.pone.0200142)
Supplement: S2 Table — (DOC) [file pone.0200142.s003.doc]

**S2 Table. Summary of overall survival information in included studies**

| **Study** | **No. of patients** | | **1-year rates** | | **2-year rates** | | **3-year rates** | | **4-year rates** | | **5-year rates** | |
| --- | --- | --- | --- | --- | --- | --- | --- | --- | --- | --- | --- | --- |
|  | **SCRT** | **LCRT** | **SCRT** | **LCRT** | **SCRT** | **LCRT** | **SCRT** | **LCRT** | **SCRT** | **LCRT** | **SCRT** | **LCRT** |
| Bujko 2006^[10]^ | 155 | 157 | 90.5% | 95.2% | 85.7% | 83% | 78.8% | 77% | 67.2% | 66.2% | NR | NR |
| Klenova A 2007^[33]^ | 51 | 33 | NR | NR | NR | NR | NR | NR | 72% | 70% | NR | NR |
| Eitta MA 2010^[15]^ | 14 | 15 | 79.3% | 84.7% | 64% | 66% | NR | NR | NR | NR | NR | NR |
| Inoue Y 2011^[32]^ | 51 | 22 | NR | NR | NR | NR | NR | NR | NR | NR | 83.4% | 83.3% |
| Ngan SY 2012^[14]^ | 162 | 161 | 89.4% | 95.1% | 89.2% | 89.6% | 82.2% | 81.7% | 78.5% | 73.9% | 74% | 70% |
| Guckenberger M 2012^[29]^ | 108 | 107 | 92.7% | 90.6% | 90.1% | 82.4% | 83.1% | 71.4% | 70.9% | 64.1% | 69% | 64% |
| Krajcovicova I 2012^[30]^ | 96 | 55 | 97.8% | 96.1% | 96.1% | 86.9% | 94% | 83% | 86.9% | 74.3% | 81% | 70% |
| Yeh CH 2012^[31]^ | 28 | 37 | 99.8% | 99.8% | 92.7% | 89.2% | 88.6% | 86.1% | 88.5% | 86.2% | NR | NR |
| Beppu N 2015^[28]^ | 104 | 61 | 98.1% | 99.8% | 96.3% | 93% | 95.1% | 93.1% | 92.8% | 87.3% | 86.9% | 87.4% |
| Kairevičė L2017^[13]^ | 68 | 72 | 95.1% | 93.1% | 84.8% | 90% | 78% | 82.4% | 70% | 80.4% | 62% | 79% |
| Abdel-Rahman O 2017^[34]^ | 241 | 186 | 95.22% | 96.19% | 88.42% | 90.13% | 80.21% | 82.59% | 68.79% | 70.93% | 60.69% | 63.26% |

SCRT: short-course radiotherapy, LCRT: long-course radiochemotherapy;

NR: not reported.
