# Supplementary material for: Preoperative short-course radiotherapy and long-course radiochemotherapy for locally advanced rectal cancer: Meta-analysis with trial sequential analysis of long-term survival data
Source: PLoS One. 2018 Jul 12;13(7):e0200142. doi: 10.1371/journal.pone.0200142 (PMC6042715; doi:10.1371/journal.pone.0200142)
Supplement: S3 Table — (DOC) [file pone.0200142.s004.doc]

**S3 Table. Summary of disease free survival information in included studies**

| **Study** | **No. of patients** | | **1-year rates** | | **2-year rates** | | **3-year rates** | | **4-year rates** | | **5-year rates** | |
| --- | --- | --- | --- | --- | --- | --- | --- | --- | --- | --- | --- | --- |
|  | **SCRT** | **LCRT** | **SCRT** | **LCRT** | **SCRT** | **LCRT** | **SCRT** | **LCRT** | **SCRT** | **LCRT** | **SCRT** | **LCRT** |
| Bujko 2006^[10]^ | 155 | 157 | 76.1% | 77.3% | 66.6% | 64.3% | 60% | 59.3% | 58.4% | 55.6% | NR | NR |
| Klenova A 2007^[33]^ | 51 | 33 | NR | NR | NR | NR | NR | NR | 66% | 68% | NR | NR |
| Eitta MA 2010^[15]^ | 14 | 15 | 81.3% | 86.7% | 61% | 83% | NR | NR | NR | NR | NR | NR |
| Inoue Y 2011^[32]^ | 51 | 22 | NR | NR | NR | NR | NR | NR | NR | NR | NR | NR |
| Ngan SY 2012^[14]^ | 162 | 161 | 86% | 83.9% | 73.3% | 76% | 68.8% | 73.4% | 62.9% | 71% | 60.6% | 66.6% |
| Guckenberger M 2012^[29]^ | 108 | 107 | 88.3% | 78.1% | 82.5% | 63.6% | 74.3% | 57.6% | 68% | 52% | 64% | 52% |
| Krajcovicova I 2012^[30]^ | 96 | 55 | 80.4% | 67.3% | 75.4% | 66% | 73% | 66% | 71.1% | 62.4% | 66% | 54% |
| Yeh CH 2012^[31]^ | 28 | 37 | 89.5% | 78.4% | 78.6% | 64.4% | 66.6% | 64.3% | 55.6% | 64.3% | NR | NR |
| Beppu N 2015^[28]^ | 104 | 61 | 89.9% | 92% | 83.8% | 78.3% | 83.8% | 78.3% | 79.8% | 78% | 79.5% | 78% |
| Kairevičė L2017^[13]^ | 68 | 72 | 85.1% | 86.4% | 68.8% | 76.4% | 59% | 75.1% | 50.8% | 70.5% | 45% | 67% |
| Abdel-Rahman O 2017^[34]^ | 241 | 186 | 95.91% | 94.57% | 87.86% | 81.08% | 78.09% | 71.57% | 70.29% | 69.01% | 66.77% | 67.09% |

SCRT: short-course radiotherapy, LCRT: long-course radiochemotherapy;

NR: not reported.
