# Supplementary material for: Preoperative short-course radiotherapy and long-course radiochemotherapy for locally advanced rectal cancer: Meta-analysis with trial sequential analysis of long-term survival data
Source: PLoS One. 2018 Jul 12;13(7):e0200142. doi: 10.1371/journal.pone.0200142 (PMC6042715; doi:10.1371/journal.pone.0200142)
Supplement: S4 Table — (DOC) [file pone.0200142.s005.doc]

**S4 Table. Summary of local recurrence information in included studies**

| **Study** | **No. of patients** | | **1-year rates** | | **2-year rates** | | **3-year rates** | | **4-year rates** | | **5-year rates** | |
| --- | --- | --- | --- | --- | --- | --- | --- | --- | --- | --- | --- | --- |
|  | **SCRT** | **LCRT** | **SCRT** | **LCRT** | **SCRT** | **LCRT** | **SCRT** | **LCRT** | **SCRT** | **LCRT** | **SCRT** | **LCRT** |
| Bujko 2006^[10]^ | 155 | 157 | 2.9% | 4.4% | 6.3% | 10.7% | 8.5% | 14.8% | 10.6% | 15.6% | NR | NR |
| Klenova A 2007^[33]^ | 51 | 33 | 1.9% | 2.9% | 5.9% | 5.9% | 6.1% | 6.1% | NR | NR | NR | NR |
| Eitta MA 2010^[15]^ | 14 | 15 | NR | NR | 14.3% | 6.7% | NR | NR | NR | NR | NR | NR |
| Inoue Y 2011^[32]^ | 51 | 22 | NR | NR | NR | NR | NR | NR | NR | NR | NR | NR |
| Ngan SY 2012^[14]^ | 162 | 161 | 2.4% | 1% | 5% | 4.4% | 7.5% | 4.4% | 7.5% | 5.7% | 7.5% | 5.7% |
| Guckenberger M 2012^[29]^ | 108 | 107 | NR | NR | NR | NR | NR | NR | NR | NR | 9% | 8% |
| Krajcovicova I 2012^[30]^ | 96 | 55 | NR | NR | NR | NR | 7.3% | 16.4% | NR | NR | 11.5% | 20% |
| Yeh CH 2012^[31]^ | 28 | 37 | 0 | 5.7% | 3.7% | 9.7% | 4% | 9.7% | 4% | 10.1% | NR | NR |
| Beppu N 2015^[28]^ | 104 | 61 | 6.1% | 2.2% | 9.4% | 9.4% | 9.4% | 9.4% | 9.4% | 9.4% | 9.4% | 9.4% |
| Kairevičė L 2017^[13]^ | 68 | 72 | NR | NR | NR | NR | 3.1% | 5.6% | NR | NR | NR | NR |

SCRT: short-course radiotherapy, LCRT: long-course radiochemotherapy;

NR: not reported.
